# Implementing cancer prevention strategies in India: progress, persistent challenges and future directions

**DOI:** 10.3332/ecancer.2025.2042

**Published:** 2025-11-19

**Authors:** Jitendra Kumar Meena, Kavita Yadav, Ravi Mehrotra

**Affiliations:** 1 National Cancer Institute (NCI), Jhajjar, Haryana 124105, India; 2 All India Institute of Medical Sciences (AIIMS), New Delhi 124105, India; 3 George Institute for Global Health, New Delhi 110025, India; 4 Emory University, 1518 Clifton Rd, Atlanta, GA 30322, USA; * Joint first authors

**Keywords:** cancer prevention, preventive oncology, India, cancer care, public health

## Abstract

Cancer is a major and growing global health crisis, placing an immense and escalating burden on the health systems of developing countries like India. Given the substantial resources demanded by curative management, a strategic investment in comprehensive cancer prevention and control becomes not merely prudent but imperative. India faces a high incidence of tobacco and lifestyle-related cancers, which are largely preventable through robust public health approaches, including tobacco control, cessation support and Human Papilloma Virus vaccination. This narrative review critically assesses the status of cancer prevention and control services in India, highlighting key achievements, inherent challenges and potential threats to progress. While India’s National Programme for Prevention and Control of Non-Communicable Diseases has initiated population-based cancer screening and expanded regional cancer centers, actual gains have been limited. This is due to implementation delays, suboptimal technical capacity and resource limitations in preventive services. Despite notable successes in areas like the cancer registry network, tobacco control legislation and the establishment of the National Cancer Grid, a consolidated and strategically implemented plan to effectively mitigate the projected increase in cancer burden remains a significant challenge.

## Background

### Cancer burden and etiology in India

Globally, cancer is the second leading cause of mortality, responsible for approximately one in six deaths worldwide. In India, the burden is particularly immense, with an estimated 2.72 million active cancer cases at any given time. The country records over an estimated 1.56 million new cases and more than 0.87 million deaths annually. global cancer observatory estimated that the incidence of cancer in India will increase to approximately 2.46 million cases by 2045 [1]. Cancers are typically multifactorial, stemming from both intrinsic and extrinsic factors. Pathogenic infections, such as Hepatitis B, C and Human Papilloma Virus (HPV), are responsible for up to 25% of cancer cases in low- and middle-income countries [2]. Specifically in India, tobacco use accounts for a substantial 33.3% of all cancers, representing 48.7% of male cancers and 16.5% among females, as reported by the Indian Council of Medical Research’s (ICMR) Hospital-Based Cancer Registry [3]. Furthermore, a high burden of cancers is linked to hazardous occupational and environmental exposures, including substances such as asbestos, chemical dyes and petrochemicals. With increasing population exposure to these risk factors, the projected annual rise in cancer cases is alarming, anticipated to reach almost 2.2 million by 2030 [4]. The most recent National Cancer Registry Program (NCRP) report estimates 1.57 million cases by 2025, a significant 12% increase from previous estimates [5]. This persistent upward trend in cancer incidence is poised to impose an increasingly insurmountable burden on India’s public health system.

### The imperative for effective cancer control in India

A concerning majority—over 70%—of cancer cases in India are diagnosed at advanced stages, which critically compromises clinical prognosis and patient survival. This late presentation is compounded by challenges within the healthcare system, including fragmented cancer diagnostic and treatment facilities, leading to poor clinical outcomes, reduced survival rates and substantial financial strain. Delays in accessing or receiving comprehensive cancer care, often averaging 2 months or more, are frequently attributed to patients’ limited awareness, a scarcity of qualified oncologists and the predominant concentration of specialised cancer centres in metropolitan areas [6].

**Figure 1.**
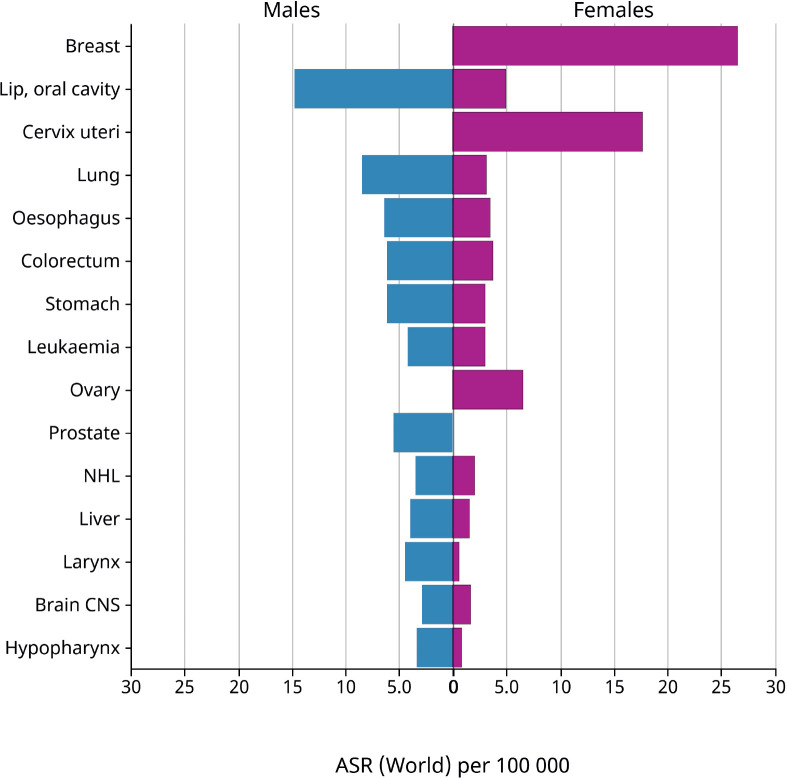
Age-standardised rate (world) per 100,000, incidence, males and females, in 2022 in India: GLOBOCAN [7].

Given the finite resources for comprehensive cancer care and the escalating number of cancer cases, there is a heightened emphasis on prioritising population-level strategies for cancer prevention and control. The National Programme for Prevention and Control of Non-Communicable Diseases (NP-NCD) aims to address cancer at the population level through organised screenings for early detection, thereby enabling disease downstaging and improving cure rates. However, the full benefits of early detection are severely constrained by the aforementioned barriers to timely diagnosis and comprehensive treatment. The projected increase in cancer burden can only be effectively mitigated through a systemic and sustained prioritisation of primordial and primary prevention strategies, complemented by robust secondary and tertiary care infrastructure (Figure 1).

#### Levels of cancer prevention

Cancer, as a chronic and complex disease, necessitates high-intensity care, dedicated resources for comprehensive diagnostics and meticulous management with long-term follow-up. The escalating burden of care underscores the critical need for a dual approach: simultaneously expanding tertiary cancer centres (TCCs) and palliative care facilities, while vigorously pursuing cancer prevention. In a resource-constrained healthcare landscape like India’s, cancer prevention emerges as a pivotal public health intervention, one that must be synergistically pursued with the expansion of cancer care resources. Understanding the distinct levels of prevention is crucial for effective strategy formulation:

### Primordial prevention

Primordial prevention aims to prevent the emergence of risk factors themselves in the first place, by addressing underlying social, economic and environmental determinants of health. This level of prevention targets root causes rather than direct risk behaviours. Examples pertinent to cancer include improving socio-economic conditions to reduce poverty-driven risk behaviours, fostering healthy urban planning to minimise environmental carcinogen exposure and implementing policies that promote equitable access to nutritious food and safe living environments. By shaping societal conditions, primordial prevention seeks to create environments where cancer risk factors are less likely to develop.

### Primary prevention

Primary cancer prevention encompasses interventions designed to prevent the exposure to or the development of, specific cancer-causing agents or conditions, thereby halting the neoplastic transformation of healthy cells or pre-cancerous conditions. These strategies are implemented before the onset of disease and include health promotion and specific protection. The array of preventive efforts may include comprehensive health counselling and education, stringent environmental controls (e.g., regulations on air pollution and occupational exposures), ensuring food product safety and universal vaccination against oncogenic pathogens like HPV and Hepatitis B Virus. Health behavioural approaches necessitate a credible source of information for effective health counselling and patient education. Furthermore, strengthening individual coping mechanisms through lifestyle and personal habits counselling, such as active tobacco cessation programs, is a critical component.

### Secondary prevention

Secondary cancer prevention consists of targeted interventions focused on the early detection and management of cancerous or precancerous processes. The aim is to diagnose disease at its earliest, most treatable stages, thereby improving prognosis and survival outcomes. Key activities include population-based screening programs and opportunistic screening for common cancers. For instance, the ‘screen and treat’ strategy for cervical cancer involves visual inspection with acetic acid (VIA) for early detection and immediate treatment of cervical intraepithelial neoplasia using thermo-ablation in a single setting. This level of prevention is crucial for downstaging disease and enabling more effective and less intensive treatment.

### Tertiary prevention

Tertiary cancer prevention refers to care activities aimed at reducing morbidity, limiting disability and improving the quality of life (QoL) among cancer patients already undergoing or post-treatment. It comprises a wide range of supportive and rehabilitative approaches addressing physical, mental, social, psychological and economic dimensions of patient well-being. This level also encompasses the establishment of risk-matched follow-up programs, prevention of cancer recurrence or the development of subsequent, distinct cancers in individuals with a history of early-stage cancer after primary treatment. Examples include tobacco cessation interventions specifically for lung cancer survivors and chemoprevention strategies in breast cancer survivors to prevent relapse or secondary cancers. The integration of comprehensive palliative care services is also a vital aspect of tertiary prevention, focusing on symptom management and holistic support.

#### Status of cancer prevention and control in India

Approximately 70% of cancer cases in India stem from potentially modifiable and preventable risk factors, with tobacco-related cancers accounting for 40%, infection-related cancers for 20% and other factors for 10% [8]. This significant burden of preventable cancers underscores the critical importance of scaling up cancer prevention services to achieve effective cancer control. In a context where the healthcare system’s curative capacity faces limitations, prioritising prevention becomes even more vital. While significant efforts are being directed towards expanding cancer diagnostics and management, commensurate resource pooling and strategic emphasis are concurrently required for cancer awareness, risk reduction and early detection.

### Policy frameworks and national programs

India’s commitment to cancer prevention is anchored in national policies and programs, primarily the National Programme for Prevention and Control of Cancer, Diabetes, Cardiovascular Diseases and Stroke (NPCDCS), launched in 2010. This program emphasises infrastructure development, human resource training, health promotion, early diagnosis and management with referral pathways for NCDs, including common cancers (oral, breast and cervical). Under NPCDCS, now NP-NCD dedicated NCD Cells have been established at various healthcare levels to foster awareness, facilitate early diagnosis, streamline referrals and ensure follow-up for these common cancers. Guidelines have been issued for initiating ‘Population-based Screening of common NCDs’ in the community, leveraging frontline workers such as Accredited Social Health Activists (ASHAs), Auxiliary Nurse Midwives (ANMs) and Community Health Officers within the existing Primary Healthcare System [9]. While the establishment of 298 District NCD Cells and 293 District NCD Clinics demonstrates a commendable foundational effort, the integration of cancer-specific interventions within this broader NCD framework has presented challenges, often leading to diluted focus and varied effectiveness in cancer outcomes. Despite its broad scope, the program’s effectiveness is limited by issues such as insufficient funding, uneven execution and low community knowledge. The program has, however, demonstrated effectiveness in certain domains, such as the management of hypertension and it has the capacity to have a greater influence through improved community involvement and strategic adjustments.

### Surveillance and epidemiological insights

Robust surveillance is fundamental to effective cancer control. The NCRP, comprising a network of 36 population-based and 236 hospital-based Cancer Registries, serves as the country’s largest source of cancer epidemiological data. Managed by the National Centre for Disease Informatics and Research (NCDIR) under the ICMR since March 2011, NCRP has expanded its scope to include other NCDs with shared etiological factors. NCDIR also conducted the National Non-communicable Disease Monitoring Survey in 2017–2018 to generate national estimates of key NCD (including cancer) risk factors like smoking, alcohol and diet, alongside health system responses. Current thrust areas include refining cancer registries, conducting Patterns of Care and Survival Studies and developing advanced software applications [10].

The National Cancer Atlas Project has provided crucial insights, notably identifying a higher incidence of cancer in India’s northeastern states, subsequently confirmed by the establishment of Population-Based Cancer Registries in those regions. This project strengthens pathology departments and provides training in cancer registration and epidemiology [11]. The National Centre for Disease Control further contributes through its India Epidemic Intelligence Service Programme, which, in collaboration with the centers for disease control and prevention, Atlanta, has been revamped to include NCDs. This program aims to build programmatic capacity in NCD risk assessment, early detection, referral monitoring and patient follow-ups, providing critical hands-on epidemiologic training to public health professionals [12].

### Cancer prevention services and infrastructure

India has made strides in integrating Preventive Oncology services within its evolving cancer care facilities, encompassing health promotion, early detection and management of precancerous conditions. The establishment of the National Cancer Institute under All India Institute of Medical Sciences, New Delhi, at Jhajjar, Haryana, serves as a nodal centre for India-centric cancer research, preventive, promotive and curative aspects of cancer care, aligning with global standards [11].

Tobacco cessation activities form an integral part of these prevention services, reflecting the high burden of tobacco-related cancers (estimated 40%–50% in India). Programs like the National Tobacco Control Programme (NTCP), now implemented across all 36 States/Union Territories, covering approximately 612 districts, have strengthened cessation services. NTCP includes provisions for setting up Tobacco Cessation Services at the District level [15]. This is often complemented by active or opportunistic screening for oral and lip cancers, facilitated by dedicated public health funds and human resources. For a comprehensive cancer care infrastructure, NP-NCD aims to establish/strengthen 20 State Cancer Institutes and 50 TCCs, with significant ‘one-time grants’ allocated for construction and equipment [9]. However, the actual impact of these infrastructure developments on equitable access and timely care for the broader population requires continuous monitoring and evaluation, given persistent regional disparities.

### Public awareness and community engagement initiatives

Generating public awareness, engagement and mobilisation for risk assessment and screening of NCDs, including common cancers, is a key mandate for Community Health Workers (ASHAs, ANMs) under the National Health Mission [9]. These frontline workers conduct regular community health education based on standard guidelines and training packages. The success of these awareness campaigns is variable but has contributed to increased knowledge in some regions, though converting this awareness into consistent behaviour change and screening uptake remains a challenge.

Advocacy campaigns through print and social media are increasingly utilised to disseminate information on cancer prevention, healthy lifestyles, tobacco control and screening. Various organisations leverage social media campaigns, particularly around health days, to amplify awareness about cancer prevention steps and risk factors. These efforts signify a growing receptiveness among the public to cancer-related health information.

### Role of Non-Governmental Organisations (NGOs) and partnerships

NGOs play a crucial complementary role in India’s cancer prevention landscape. Organisations such as the Indian Cancer Society and Charutar Arogya Mandal actively support early cancer detection activities through various centres and mobile camps, often focusing on underprivileged populations while also striving to provide multidisciplinary cancer care [14]. Other NGOs like Udhavum Ullangal Public Charitable Trust contribute to education and healthcare, specifically in cancer prevention and patient rehabilitation. Specialised NGOs cater to specific populations, such as Cuddles Foundation and St. Jude India Childcare Centres for children and Women’s Cancer Initiative – Tata Memorial Hospital for women’s health [14]. Their localised understanding and community roots enable innovative, context-specific outreach models, complementing broader government efforts.

Public-Private Partnerships (PPPs) are also emerging as a model for cancer care. The Tata Memorial Centre (TMC), a Grant-in-Aid Institution, collaborates with Tata Trusts and operates centres across nine locations, providing highly subsidised or free treatment to a significant proportion of poor cancer patients. TMC also coordinates the National Cancer Grid (NCG), a publicly funded network of over 250 cancer centres established in 2012. NCG’s primary mandate is to ensure uniform standards of patient care across India by promoting evidence-based prevention, screening and management guidelines [13, 16]. Innovative models like the ‘Distributed Cancer Care Model’ proposed by some state governments (e.g., Assam) aim to improve coverage in high-burden regions by developing a tiered system of apex referral centres (L1), comprehensive hospitals (L2) and diagnostic/day care centres (L3) in collaboration with private entities [17].

While the private health sector plays a dominant role in tertiary cancer care, largely concentrated in urban centres, its engagement in preventive oncology services like mammography, gynaecological screenings and HPV immunisations is growing among large corporate hospital chains. However, broader cancer awareness, health promotion and population-based screening activities within the private sector are still predominantly led by private trusts and NGOs.

#### Cancer prevention research landscape and gaps

Despite the escalating cancer burden in India, a significant gap persists in the realm of cancer prevention research, particularly concerning applied, community-based and translational studies. While numerous small-scale studies are regularly published, their impact on population policies and clinical practice often remains limited. This lag in cancer prevention research can be attributed to a confluence of factors, including inadequate training in preventive oncology research, insufficient research collaborations, limited availability and sharing of crucial resources (such as epidemiologists, statisticians and well-equipped laboratories) and a scarcity of well-maintained population-based cohorts.

The current landscape of cancer research in India, while robust in certain areas, needs strategic redirection to bolster prevention. Some of the salient initiatives taken by the Government of India are:

### National Institute of Cancer Prevention and Research (NICPR, ICMR)

The NICPR, a premier ICMR research institute, plays an instrumental role in cancer prevention. It actively promotes health through its clinic, offers online courses for screening common cancers (breast, oral and cervical) and hosts the National Tobacco Testing Laboratory. NICPR’s designation as the WHO Framework Convention on Tobacco Control (FCTC) knowledge hub for smokeless tobacco (SLT) underscores its strategic importance. Furthermore, NICPR maintains a comprehensive, public-facing website disseminating updated information on common cancers and their risk factors in accessible language, thereby contributing to general cancer awareness.

### Government research grant initiatives

The Department of Biotechnology and Department of Science & Technology (DST), Government of India, offer competitive research grants aimed at fostering collaborations and bringing together cancer experts. These governmental funds support diverse categories of cancer research, spanning epidemiology, molecular biology, genomics, proteomics, epigenetics, genome-wide association studies, biostatistics, imaging and studies utilising cell lines and animal models [18]. While such initiatives have supported cancer prevention research, their scope has been somewhat limited compared to the vast national need. Over time, dedicated cancer grants, such as those offered by the India Cancer Research Consortium, have begun to include specific funding options under the thematic areas of cancer prevention and epidemiology, indicating a growing, albeit nascent, recognition of this priority [19].

### Translational and basic research focus

A predominant focus in India’s translational and laboratory-based research remains on the diagnostic and therapeutic aspects of cancer disease. For instance, a notable study in India demonstrated the efficacy of an ultra-low dose of the immunotherapy drug nivolumab (Opdivo) in extending the lifespan of individuals with advanced head and neck cancer, potentially making advanced treatments more affordable and accessible in low- and middle-income countries [20]. While such advancements in treatment are crucial, a parallel emphasis is needed on cancer prevention research. This includes promoting studies on the translation of research findings into clinical practice within the Indian population, particularly for the development of indigenous HPV vaccines, the identification of novel biomarkers for early cancer detection and the creation of accessible tests for precancerous conditions in oral or cervical cancers.

### Integrative approaches with traditional medicine systems

The integration of traditional medicine systems alongside conventional cancer prevention and therapeutic interventions (modern medicine) is gaining traction in India as a comprehensive, individual-centred, evidence-based approach. Medicinal herbs and their derivative phytocompounds are increasingly recognised as valuable adjuncts in cancer treatments. Numerous clinical studies have reported the beneficial effects of herbal medicines on patient survival, immune modulation and QOL when used in conjunction with conventional therapeutics [21]. The three pillars of complementary medicine—lifestyle modifications, mind-body practices and the use of natural products—hold significant potential for cancer prevention and for enhancing QOL and even treatment response in cancer patients when combined with modern medical practices [22]. Further research into their efficacy and integration within national prevention strategies is warranted.

#### Key achievements and progress in cancer prevention in India

India has demonstrated significant commitment and achieved notable successes across various facets of cancer prevention and control, driven by legislative frameworks, national programs and the collective efforts of governmental and non-governmental entities. These achievements lay a crucial foundation for future advancements, even amidst persistent challenges.

### Robust tobacco control legislation and programs

India’s tobacco control efforts have been substantially fortified by the enactment of the Cigarettes and Other Tobacco Products (Prohibition of Advertisement and Regulation of Trade and Commerce, Production, Supply and Distribution) Act (COTPA) in 2003, followed by the ratification of the WHO FCTC in 2004. This commitment led to the formulation of the NTCP in 2007–2008. These legislative and programmatic interventions have been fairly successful in reducing tobacco consumption rates, as evidenced by reports from the Global Adult Tobacco Survey (GATS) and Global Youth Tobacco Survey. Adult tobacco use showed progress, declining from 34.6% in GATS-1 (2009–2010) to 28.6% in GATS-2 (2016–2017), indicating a notable 17% relative reduction. This drop suggests growing awareness, cessation efforts and policy impact among adults. However, despite these gains, progress often falls short of anticipated public health goals, particularly in limiting access for teenagers and young adults, with recent studies highlighting compliance violations in a significant proportion of schools [23]. Sustained and successful enforcement of COTPA, NTCP and FCTC necessitates the active engagement of civil society to increase public awareness, garner support for legislation and involve communities in monitoring and reporting violations. The WHO MPOWER package [24, 25] provides a comprehensive framework that guides these effective interventions to decrease tobacco demand and track the status of the tobacco epidemic globally, in which India has made progress.

### Alcohol policy and state-specific bans

Alcohol use contributed a substantial 6.6% to the total cancer Disability-Adjusted Life Years (DALYs) in India in 2016, making it the second leading risk factor after tobacco (10.9%), according to the Global Burden of Disease [26]. In India, the authority for alcohol sale taxation and Minimum Legal Drinking Age) rests with State government excise departments, allowing states to control pricing, safety, quality and prevent illicit sales [27]. Notably, several states and union territories, including Bihar, Gujarat, Mizoram, Nagaland and Lakshadweep, have implemented complete bans on alcohol sales. Mizoram, for instance, reinstated its ban in 2019, reflecting a renewed policy commitment [28]. While the Cable TV Networks Amendment Bill 2000 prohibits liquor and tobacco advertisements, surrogate advertising by alcohol companies remains a significant challenge, with evidence suggesting its role in increasing alcohol use among adolescents [29].

### Strengthening packaging regulations

India has made considerable progress in enhancing health warnings on tobacco products. The country has implemented a maximum of 85% pictorial health warnings on all tobacco products, a significant step towards discouraging consumption [30]. Efforts are also underway to introduce plain packaging, further aimed at stripping tobacco packs of their glamour and appeal. While Front-of-Package Labels on food items are crucial for helping consumers identify nutrients of concern (sugar, saturated fat and sodium) and make informed dietary choices to prevent diet-related NCDs [31], compliance with health warning labels on food products and alcohol in India remains poor. Learning from international examples, such as Ireland’s adoption of health warning labels on alcohol products, could provide a model for effective implementation in India through robust market surveillance [32].

### Food safety and anti-adulteration measures

The Food Safety and Standards Authority of India, established under the Food Safety and Standards Act of 2006, plays a pivotal role in the safety monitoring and regulation of food products. Recognising that unsafe food can pose serious health risks, including fatal diseases like cancer, the Act provides a consolidated channel for food safety. Importantly, amendments to the Prevention of Food Adulteration Act of 1990 were made to include statutory warnings regarding the harmful health effects of tobacco and gutka products, which were categorised as food items [33]. Furthermore, in 1992, the Drugs and Cosmetics Act of 1940 (Amendment) banned the use of tobacco in all dental products, reflecting a proactive approach to address carcinogens in consumer goods [34].

### Environmental pollution control initiatives

The Ministry of Environment, Forest and Climate Change is mandated to monitor and preserve the environment to ensure public health and well-being. While the International Agency for Research on Cancer regularly assesses environmental carcinogens, there remain gaps in India’s environmental monitoring and epidemiological reviews specific to carcinogenicity from hazardous industries and their impact on vulnerable populations. ICMR’s National Institute for Research in Environmental Health and National Institute of Occupational Health are actively developing projects to review the cancer hazard posed by environmental pollution. Such comprehensive reviews and detailed action plans are poised to profoundly impact the health status of India’s future generations. The Indian Space Agency, under the DST, holds potential to support geo-spatial mapping of cancer incidence alongside environmental pollution in vulnerable populations, facilitating targeted interventions like the relocation of non-compliant polluting industrial units [35].

### Protection of youth and children from carcinogens

As a critical primordial prevention strategy, the Government of India has introduced several legislations aimed at protecting young citizens from carcinogens. The Juvenile Justice Act of 2015, for example, imposes rigorous imprisonment and fines on anyone providing intoxicating liquor, narcotic drugs, tobacco products or psychotropic substances to children without a medical prescription [36]. Beyond punitive measures, initiatives like Fit India and Khelo India promote physical activity and sports, targeting young people to foster healthy lifestyles. Furthermore, a series of excise tax increases on tobacco products—ranging from 22% for bidis, 52.7% for cigarettes and 63.8% for SLT—have been implemented to reduce access to these carcinogenic substances among the youth [37].

### Advancing HPV vaccination for cervical cancer prevention

A highly promising intervention for preventing cervical cancer is vaccination against HPV. HPV types 16 and 18 are collectively responsible for approximately 70% of all invasive cervical cancer cases worldwide. India has made a significant leap towards cervical cancer elimination with the upcoming indigenous quadrivalent HPV vaccine, ‘CERVAVAC,’ developed by the Serum Institute of India (SII) Private Limited. This affordable and cost-effective vaccine is an outcome of a partnership between DBT, Biotechnology Industry Research Assistance Council and the Bill and Melinda Gates Foundation, reflecting a collaborative effort towards ‘Atmanirbhar Bharat’ (self-reliant India) in health [38]. Such initiatives are pivotal in aligning India’s efforts with WHO’s global cervical cancer elimination target of immunising 90% of girls by the age of 15 by 2030.

#### Roadblocks and implementation challenges for cancer prevention

Despite significant policy intentions and foundational efforts, India’s cancer prevention and control strategy encounters substantial roadblocks that limit its overall effectiveness. As the nation increasingly grapples with the dual burden of communicable and NCD, a disproportionate allocation of resources often favours the establishment of cancer treatment facilities. This pervasive ‘treatment-centricity’ within the cancer care model, unfortunately, marginalises both preventive and palliative care, curtailing resources and opportunities for impactful population-level interventions.

### Gaps in primary and secondary prevention implementation

A glaring missed opportunity lies in the delayed and fragmented implementation of widespread HPV vaccination, which holds immense potential for drastically reducing cervical cancer cases and associated mortality. While an indigenous vaccine is on the horizon, the lack of a comprehensive, universal public vaccination campaign has left a critical vulnerability. Time-bound rollout of HPV vaccines is pertinent in the country due to the high disease burden and mortality on the global comparison. The vaccine offers a highly effective way to prevent cervical cancer by targeting the prevalent HPV strains and interrupting the chain of transmission.

Furthermore, scaling up screening and early management for cervical cancer remains challenging due to the invasive and time-consuming nature of the Pap test, which is not easily scalable for population-wide use in India. While the ‘screen and treat’ model using VIA offers a more practical alternative, its widespread adoption is still limited by the need for trained personnel and dedicated infrastructure. A systemic approach is needed that leverages frontline health workers and integrates these screening methods into existing primary healthcare services to ensure broad coverage and consistent follow-up. This challenge is further exacerbated by new recommendations for first-line HPV DNA-based cervical cancer screening, which, despite being pragmatically superior, are difficult to implement broadly in low-resource settings due to limited infrastructure, poor acceptance and cost barriers.

### Industry interference and addiction support deficiencies

The relentless lobbying and sophisticated marketing tactics employed by the tobacco industry pose a continuous and formidable challenge to subverting tobacco control legislation. Public health agencies often must dedicate significant litigation and advocacy resources simply to counter these powerful forces. Tobacco consumption, both in smoked and smokeless forms, remains a pervasive menace in India, and its effective control is paramount for significantly limiting the cancer burden. A critical lacuna in the current strategy is the severe lack of accessible and comprehensive de-addiction and support services for individuals seeking to quit tobacco and alcohol use once initiated. This deficiency creates a significant barrier to behaviour change, undermining prevention efforts.

### Human resource and capacity deficits

A fundamental roadblock in cancer prevention efforts is the pervasive deficiency of adequately trained human resources and robust training infrastructure in cancer prevention and preventive oncology. This impacts every level, from academic teaching to clinical practice and research. The limited technical capacity in preventive medicine, whether within medical academia or applied research settings, severely hampers the country’s cancer control efforts. This gap is particularly significant as the NP-NCD program’s scope expands to include other diseases like Non-Alcoholic Fatty Liver Disease and Chronic Kidney Disease, further straining already limited resource.

### Resource allocation and equitable access

Beyond human resources, equitable allocation of financial and infrastructural resources remains a significant challenge. While national programs aim to expand cancer care, the distribution of facilities and specialised personnel is often skewed towards urban centres, leaving vast rural and remote populations underserved. This creates significant access barriers to both early detection and advanced treatment, exacerbating health disparities.

### Gaps in data utilisation and research translation

Despite the existence of national registries and surveillance programs, the effective translation of epidemiological data into actionable population-level policies and clinical practices remains an area for improvement. The limited conduct of large-scale, applied and community-based cancer prevention research further restricts the evidence base needed for developing context-specific interventions. Challenges in research collaboration and resource sharing impede the development of indigenous solutions and the rigorous evaluation of program effectiveness.

### Stakeholder level analysis of existing gaps:

1. Central Government / National Health Authorities

The government’s role includes policy formulation, funding under NP-NCD and strengthening cancer registries. However, cancer prevention is often sidelined, with limited budgets, weak monitoring, poor intersectoral coordination, partial registry coverage and so on. Delays in HPV vaccine integration in national immunisation program is one of the major gaps in cancer prevention despite development of indigenous vaccine.

2. State Governments / State Health Department

State governments are expected to adapt national cancer guidelines to local needs, ensure infrastructure, trained workforce and reliable supply chains, and monitor district-level performance. However, gaps persist with uneven prioritisation across states, shortage of trained oncology staff and weak referral pathways, leading to poor continuity from screening to treatment.

3. District Health Administration

District health administrations are responsible for implementing oral, breast and cervical cancer awareness and screening through primary healthcare workers (PHCs) and Community Health Centers and ensuring referral linkages. Yet, the screening coverage remains below 5%, with poor community participation, high dropouts, weak data quality and action and limited Information, Education and Communication efforts undermining comprehensive cancer control.

4. ASHAs, ANMs, PHC Staff

Frontline health workers are expected to mobilise communities, educate on risk factors, conduct basic cancer screening and ensure follow-up of screened individuals. However, training gap, heavy workload from competing health priorities and lack of performance-based incentives reduce effectiveness and weaken their role in cancer control.

5. TCCs and Academic Institutions

TCCs are expected to deliver advanced diagnostics and treatment, build capacity of peripheral facilities, drive contextual research and innovation and strengthen cancer registries. Yet, most are urban centric and reach out through tele-oncology and district training is weak. Oncology research remains treatment-focused, with prevention and implementation research underprioritised.

6. Private Sector and NGOs

The private sector is expected to complement public services through awareness, screening, treatment, innovative delivery models and Corporate Social Responsibility-funded cancer programs. However, engagement is often fragmented and project-based, with services concentrated in urban markets, equity concerns and poor data sharing for epidemiolocal surveillance and case tracking. However, there have been instances where PPPs have shown promise of expanding preventive services, i.e., mobile medical units, diagnostic services, innovation hubs and so on.

7. Community and Civil Society

Community and civil society groups are expected to drive demand for cancer screening and vaccination, provide peer support to patients and advocate for policy change. Yet, widespread myths, stigma and fatalism hinder participation, while the absence of strong survivor or patient networks limits organised advocacy and policy influence.

Overall, Cancer control in India faces cross-cutting systemic gaps, including underfunding within NP-NCD, shortage of oncology specialists and trained frontline workers, poor community participation and weak referral pathways between primary, district and tertiary care. Incomplete registries and poor private sector reporting limit evidence-based planning, while urban-centric services undermine equity and timely access for rural populations.

#### Current status and trends in cancer burden in India

Recent data underscore the persistent and growing challenge of cancer in India. According to the NCRP, the estimated number of new cancer cases in India was 1,461,427 in 2022, with projections indicating a rise to 1,569,793 by 2025—a 12.8% increase in just 3 years. The crude incidence rate stands at 100.4 per 100,000 population, and one in nine Indians is likely to develop cancer in their lifetime. The most common cancers are lung cancer in men and breast cancer in women, while lymphoid leukaemia is the leading site among children (0–14 years) [39] (Table 1).

Notably, the age-standardised incidence rates are highest in the North-Eastern states, with districts like Aizawl and Papum Pare reporting rates of 269.4 and 219.8 per 100,000, respectively [40]. Despite increased awareness and screening efforts, India continues to face a high proportion of late-stage diagnoses. Only 29% of cancers are detected at an early stage, with just 15% of breast, lung and 33% of cervical cancers diagnosed in stages I and II. This late detection contributes to a high mortality-to-incidence ratio and catastrophic health expenditures and wide economic losses [41].

While India has made progress in expanding cancer registries and screening infrastructure, the persistently low early detection rate and high late-stage presentation highlight ongoing gaps in public awareness, access and health system responsiveness. The economic and social impact of cancer is compounded by regional disparities and the high out-of-pocket costs for diagnosis and treatment.

**Figure 2.**
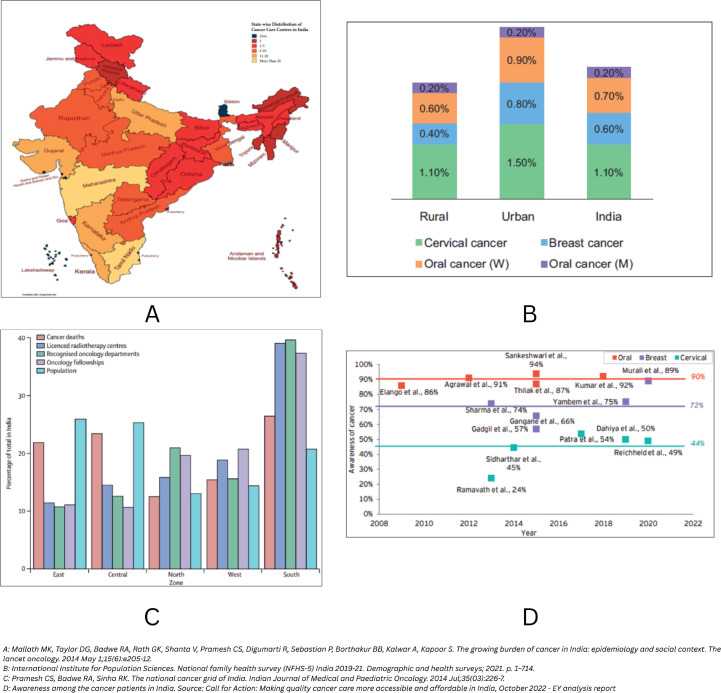
Cancer burden (A), screening uptake (B), oncology resources (C) and cancer awareness among patients (D) in India [41].

Comparative analysis with global counterparts reveals that India’s detection rates for early-stage cancers lag significantly behind those of China, the UK and the USA [4]. This underscores the need for further investment in community engagement, robust screening programs and health system strengthening (Figure 2).

#### Recommendations and the path forward

To effectively mitigate India’s growing cancer burden, a multi-pronged strategy is essential, focusing on the systemic improvements identified in this review. To summarise, the following issues need to be prioritised, in addition to others:

### Prioritise screening and early detection

Early detection and screening programs require the development of clear guidelines, manuals and decision-making tools. Investments in Preventive Oncology resources are vital, as is a robust system for follow-up and quality assurance. Quality of care and population coverage must be emphasised, with indicators measuring the effect and quality of care in terms of sensitivity, specificity, incidence mortality and referral rates. Potential barriers to screening, such as cancer stigma, fatalism and gender inequities, need to be better understood through quantitative and qualitative studies to inform policies.

### Health policy and economics

Health economic analyses of cancer prevention approaches will be key for optimising resource allocation and prioritisation. Health Technology Assessments and Schemes like Ayushman Bharat and e-Sanjeevani can be leveraged to support these efforts. There is a heightened need for dedicated financial pooling and resource investment in the field of cancer prevention services and research.

### Leveraging digital health technology

Digital health technology can revolutionise cancer prevention in India through expanding access to screening, remote consultations and personalised care and navigation pathways. Artificial Intelligence tools, i.e., point of care diagnostics, telemedicine and mobile health apps improve access in rural areas, reduce delays and support case tracking and monitoring. In the wake of limited oncology infrastructure it is important to develop digital public infrastructure for development of tech-driven models for scalable and equitable healthcare solutions.

### Decentralising cancer care

Establishment of Day Care Cancer Centres across District Hospitals is a transformative step towards equitable cancer care aiming to bring oncology services closer to people. This would reduce travel burden on patients, improve treatment compliance, facilitate early diagnosis and timely treatment while decongesting tertiary centres and save resources. Effective community level interventions are backbone of population level epidemiological risk mitigation and control of cancer epidemic.

### Building on success

Finally, a comprehensive strategy must build on proactive steps already taken by the Indian government. These include expanding preventive oncology services, ratifying strict anti-tobacco COTPA laws, implementing maximum warning labels on tobacco products, pre-emptively banning Electronic Nicotine Delivery Systems and developing indigenous HPV vaccines. These actions are crucial for achieving the mission of population cancer prevention and control on a mass level.

## Conflicts of interest

The authors have no conflicts of interest.

## Funding

The study did not receive external funding.

## Figures and Tables

**Table 1.** Estimated cancer incidence, mortality and its economic impact in India, 2020–2025 [40].

Year	New cases (NCRP)	Crude incidence rate (per 100,000)	Estimated deaths	Economic burden (USD)	Estimated DALYs
2020	1,392,179	97	900,000	$11B	~26.1 million
2022	1,461,427	100.4	900,000	$18–20B	~26.7 million
2025	1,569,793	105	1,000,000	$36–40B	~29.8 million
